# Introduction to Special Issue “The 11th International Retroviral Nucleocapsid and Assembly Symposium”

**DOI:** 10.3390/v12111243

**Published:** 2020-10-31

**Authors:** Mark C. Williams, Akira Ono

**Affiliations:** 1 Department of Physics, Northeastern University, Boston, MA 02115, USA; 2 Department of Microbiology and Immunology, University of Michigan, Ann Arbor, MI 48109, USA

The 11th International Retroviral Nucleocapsid and Assembly Symposium was held August 15–17, 2019, on the campus of Northeastern University. The meeting consisted of 40 oral presentations and a poster session while providing a stimulating environment to advance our understanding of retroviral assembly and replication and promoting interactions between young scientists and prominent researchers. The presentations covered a range of topics related to retroviral replication and assembly, including biophysical and structural studies, the roles of Gag, NC, RNA, and membranes in retroviral replication and assembly, interactions with cellular factors, virus assembly and budding, and therapeutic strategies. As an outcome of the meeting, this Special Issue was organized. Due to the pandemic, deadlines for the Special Issue were extended, and the issue was completed in October 2020.

The eleven manuscripts published as part of this Special Issue reflect the breadth of topics covered by the symposium, ranging from fundamental biophysical studies of the molecular interactions involved in retroviral replication and assembly to the characterization of complex cellular interactions involved in these processes. Studies of molecular biophysics and biochemistry included a study of the dimerization of the Rous Sarcoma Virus genomic RNA [1], comparison of the nucleic acid chaperone activity and RNA destabilization of HIV-1 Gag and its cleavage product NCp7 [2], and dissection of the contributions of different domains within the HIV-1 Gag polyprotein to specific and nonspecific interactions with RNA [3]. Continuing the theme of critical RNA structural transitions and interactions, another manuscript describes a new approach to the 3D modeling of viral regulatory RNA [4]. These studies shed new light on or describe methods to study critical retroviral replication processes in vitro at the molecular level. Additional studies probed retroviral replication in the context of subcellular structures. They include a study of roles played by an RSV NC basic sequence in RNA nuclear export and packaging specificity [5] and an investigation of a determinant for recruitment of TSG101, a host ESCRT pathway protein, to HIV-1 assembly sites at the plasma membrane [6]. Another study zeroed in on the order and frequency of recruitment of other ESCRT proteins to each single virus assembly site, dissecting the complex molecular mechanism of particle release [7]. Beyond retroviruses, a manuscript describing bioinformatics and meta-analyses conceptualizes how most viruses induce liquid–liquid phase separation to form nucleocapsid protein biomolecular condensates towards virus particle formation [8]. Finally, three review articles cover recent findings on the molecular details of how ESCRT pathway proteins facilitate retroviral particle release [9], lipid- and tRNA-regulated HIV-1 Gag localization to the plasma membrane and its effects on transmembrane protein distribution [10], and the mechanism of membrane microdomain phase separation as determined from live cell imaging of the retroviral assembly process [11]. Overall, these studies demonstrate how interactions between scientists with different areas of expertise lead to the application of new methods, which in turn yields significant new insights into critical processes in retroviral replication and assembly.
